# Editors’ Book Table

**Published:** 1875-02

**Authors:** 


### Editors' Book Table.

[NOTE.—All works reviewed in the pages of the CHICAGO MEDICAL JOURNAL may be found in the extensive stock of W. B. KEEN, COOKE & CO., whose catalogue of Medical Books will be sent to any address upon request.]

THERAPEUTICS AND MATERIA MEDICA. A Systematic Treatise on the Action and Uses of Medical Agents, including their Description and History. By *Alfred Stille, M.D.*, Professor of the Theory and Practice of Medicine and of Clinical Medicine in the University of Pennsylvania, etc., etc. Fourth edition, thoroughly revised and enlarged; in two volumes. Philadelphia: Henry C. Lea. 1874.

From the publication of the first edition "Stille's Therapeutics" has been one of the classics; its absence from our libraries would create a vacuum which could be filled by no other work in the language, and its presence supplies, in the two volumes of the present edition, a whole cyclopædia of therapeutics.

The present is simply an improvement upon the former edition, not in plan and scope of its matter, nor in the style of its presentation, for the breadth of the one and the elegance of the other scarcely admitted improvement, but in the additional matter, whose introduction has been necessitated by the advances in science, and also in the evidences of careful revision to which the whole has been subjected.

The new matter added constitutes about two hundred and fifty pages, of which the article upon Electricity covers a considerable portion. The author is a most uncompromising champion of the exclusive value of clinical experience as the basis for the determination of the therapeutic application of medicine, and considers the deduction of the "therapeutical uses of medicines from their physiological action" as a "mischievous error,"—which opinion we beg leave to deprecate as altogether too exclusive, as clinical medicine without physiological experiment would lack one of its most efficient guides, one of its sources of enlightenment, without whose aid it would grope doubtfully in the darkness of empiricism; and physiological experiment without the endorsement of clinical observation, would lack its only reliable certificate of therapeutic usefulness.

The language and style of the work evinces the professional erudition and scholastic accomplishment of the author, and, aside from its scientific and practical value, will serve as a model for professional authors who have yet to learn that the medical man is not the worse physician for being the accomplished scholar. H.

PAMPHLETS RECEIVED.

A Brass Ring Lodged in the Larynx for Four Years Removed by Sub Hyoidean Laryngotomy. Cure, by *Geo. M. Lefferts, M.D.*, Surgeon to the New York Eye and Ear Infirmary. 1875.

Transactions of the American Otological Society. Seventh Annual Meeting. Newport, R. I., July 15, 1874.

How do Spermatozoa Enter the Uterus? By *Joseph R. Beck, M.D.*, Fort Wayne, Indiana. Reprinted from the American Journal of Obstetrics for November, 1874. New York: Wm. Wood & Co. 1874.

On Reflex Irritations throughout the Genito-Urinary Tract, Resulting from Contraction of the Urethra at or near the Meatus Urinarius, Congenital or Acquired. By *Fessenden N. Otis, M.D.* 1875.

Letter to the Committee of Citizens of the proposed Schuylkill Drove Yard and Abattoir. By *Jno. H. Rauch, M.D.*, Treasurer of the American Health Association, late Sanitary Superintendent of Chicago, Ill. Philadelphia. 1874.

Contributions to the Annals of Medical Progress and Medical Education in the United States, before and during the War of Independence. By *Joseph Toner, M.D.*, Washington: Government Printing Office. 1874.

REPORT OF THE HEALTH OFFICER OF THE CITY AND COUNTY OF SAN FRANCISCO, for the Fiscal Year ending June 30, 1874. By *Henry Gibbons, Jr.*, Health Officer. San Francisco. 1874.

DYSMENORRHEA—A paper read before the Sacramento Society for Medical Improvement, June 16, 1874, by *J. E. Oatman, M.D.*

MEDICO-LEGAL SOCIETY OF THE CITY OF NEW YORK. Third Inaugural Address of *Clark Bell, Esq.*, Nov. 27, 1874.

SEVENTH ANNUAL REPORT, with the Constitution and List of Subscribers of the Toronto Eye and Ear Infirmary. 1874.

FREE PHOSPHORUS IN MEDICINE, WITH SPECIAL REFERENCE TO ITS USE IN NEURALGIA; A Contribution to Materia Medica and Therapeutics. By *J. Ashburton Thompson*, Surgeon at King's Cross to the Great Northern Railway Company, Surgeon Accoucheur to the Royal Maternity Charity. London: H. K. Lewis, 136 Gower street, W. C. 1874.

THE DISEASES OF THE STOMACH; being the third edition of the "Diagnosis and Treatment of the Varieties of Dyspepsia." Revised and Enlarged. By *Wilson Fox, F.R. C.P., F.R.S.*, Physician Extraordinary to her Majesty the Queen, etc. Philadelphia: Henry C. Lea. 1875.

JOURNALS RECEIVED.

The Atlanta Medical and Surgical Journal—December, 1874, January, 1875.
The American Medical Weekly, Louisville—December 26, Jan. 2, 9, 16.
The American Journal of Insanity—January, 1875.
The American Practitioner, Louisville—January, 1875.
The American Journal of Medical Sciences—January, 1875.
L'Anatomie et de la Physiologie Journal de l'homme et des Animaux—Charles Robin. Paris. 1875.
The Boston Medical and Surgical Journal—Jan. 1, 7, 14, 21.
The Boston Journal of Chemistry—January, 1875.
The Clinic, Dec. 26, Jan. 2, 16, 23.
The Cincinnati Lancet and Observer—January, 1875.
The Canada Medical and Surgical Journal—January, 1875.
The Druggists' Circular—January, 1875.
The Detroit Review of Medicine and Surgery—January, 1875.
The Dental Cosmos—January, 1875.
The Indiana Journal of Medicine—Dec., 1874, Jan., 1875.
The Kansas City Medical Journal—December, 1874.
The London Lancet—December, 1874, Jan., 1875.
The Laboratory, Boston—December, 1874.

The Medical Times, Philadelphia—Dec. 26, Jan. 2, 9, 16, 23.
The Medical and Surgical Reporter, Philadelphia—Dec. 26, Jan. 2, 9, 16, 23.
The Medical Record, New York—January 2, 9, 16, 23.
The Medical Examiner, Chicago—January 1, 15.
The Medical Press and Circular—London, Dec. 23, 1874.
The Missouri Clinical Record—St. Louis, January, 1875.
The Medical Herald, Kansas City, January 1, 1875.
The Medical News and Library, Philadelphia—January, 1875.
The Monthly Abstract of Medical Science—January, 1875.
The Nashville Journal of Medicine and Surgery—January, 1875.
The New York Medical Journal—January, 1875.
The Ohio Medical and Surgical Reporter—January, 1875.
The Psychological and Medico-Legal Journal—December, 1874, January, 1875.
The Practitioner—Dec., 1874, Jan., 1875.
The Pharmacist, Chicago—January, 1875.
The Pharmacal Gazette, Nashville, Tenn.—Jan. 6 and 13.
The Pacific Medical and Surgical Journal—January.
Le Progres Medical, Paris—Nos. 46, 47, 48, 49, 50, 51, 52.
The Peninsular Journal, Detroit—January, 1875.
The Practitioner.
The Richmond and Louisville Medical and Surgical Journal—December.
El Repertorio Jalisciense, Guadalajara—Nos. 3, 4, 5.
The Sanitary Journal, Toronto—January, 1875.
The Southern Medical Record, Atlanta—December, 1874.
The St. Louis Medical and Surgical Journal—January, 1875.
The Virginia Medical Monthly—January, 1875.

### *Meeting of the Alumni of the Albany Medical College.*

The Second Annual Meeting of the "Association of the Alumni of the Albany Medical College" was held in the City of Albany on Tuesday, Dec. 22nd. The following officers were elected for the ensuing year:

*President*—Dr. Jno. H. Beech ('41), Michigan. *Vice-Presidents*—Drs. Alson D. Hull ('41), New York; B. A. Mynderse ('53), New York; Alex. Shiland ('53), New York; Sol. Van Etten ('53), New York, and Charles L. Spencer ('57), Massachusetts. *Secretary*—Dr. Willis G. Tucker ('70), New York. *Treasurer*—Dr. G. L. Ullman ('71), New York. *Executive Committee*—Drs. H. D. Didama, ('46); William S. Going, ('41); James H. Scoon, ('49); James G. Bailey, ('53); M. H. Burton, ('53); Jno. H. Hill, ('53); Chas. H. Burbeck, ('59); A. P. Ten Eyck, ('66); J. H. Blatner, ('72); Oscar Myers, ('73).

The address of the retiring President, Prof. Didama, of the Syracuse University, was listened to with much interest. In the evening, the Association partook of its annual supper at the Delavan House, about one hundred and forty graduates being present.

